# Under the Surface: A Rare Case of the En Coup De Sabre Subtype of Linear Morphea in the Rio Grande Valley

**DOI:** 10.7759/cureus.114212

**Published:** 2026-08-09

**Authors:** Miguel Lopez, Jared Hensley, Maria Villegas

**Affiliations:** 1 Dermatology, University of Texas Rio Grande Valley School of Medicine, Edinburg, USA

**Keywords:** cellcept (mycophenolate mofetil), en coup de sabre morphea, hydroxychloroquine treatment, morphea variants, plaque formation, skin ulcers

## Abstract

Localized scleroderma, also referred to as 'morphea,' is a rare autoimmune disorder that affects the superficial layers of the skin and is characterized by both erythematous and bruise-like lesions with signs of central skin tightening or sclerosis; deeper lesions may be identified as edematous, erythematous plaques in the absence of well-defined borders. A subtype named en coup de sabre (ECDS) exists in the classification of linear morphea and presents with a linear induration on either the face or scalp. Our patient is a 21-year-old Hispanic female initially residing outside of the South Texas region who experienced an onset of skin thickening and blisters on her right leg in April of 2023 after returning from Europe.

Initially, she was diagnosed as having stasis dermatitis, but due to progression of her cutaneous symptoms, a skin biopsy was performed in September of 2023, which confirmed a diagnosis of morphea. After 10 months of initial treatment with a dermatologist outside of South Texas, she presented to the UT Health Rio Grande Valley (UTRGV) dermatology specialty clinic (Edinburg, TX, USA) with new and evolving skin plaques across her body, along with a forehead lesion that was consistent with the ECDS subtype of linear morphea. During this time, care was also formally established with local rheumatology and wound care specialists who had a pivotal role in the management of her condition. This case discusses the variability in clinical presentation of morphea, its mixed nature of lesions, its progression, its varied response to medical therapy, and the need for multidisciplinary management.

## Introduction

Based on a 2024 cohort study using United States health system data, the incidence rate of morphea was estimated at approximately one case per 10,000 patients per year. This was four times higher than previous estimates from a predominantly white population in Olmsted County, Minnesota [1]. The demographic distribution of this rare autoimmune condition consists of an increased prevalence in female patients [2,3]. Disease onset occurs in a bimodal distribution: either in childhood or midway through adulthood, with reported mean ages of 10 and 45, respectively [4]. However, this does not exclude the possibility of disease onset and diagnosis in early adulthood, such as in our patient. This case presents a 21-year-old Hispanic female patient who developed a severe cutaneous rash with biopsy findings consistent with morphea. The longevity and severity of her condition highlight a need for the use of different immunotherapies, close follow-up, and collaboration between dermatologic and rheumatology specialists.

This report was presented as a poster presentation at the Texas Dermatologic Society Spring 2025 Annual Meeting on April 4th, 2025, in Galveston, Texas, USA.

## Case presentation

Our patient is a 21-year-old Hispanic female initially residing outside of the South Texas region. She was vacationing in Europe at the time of symptom onset and had no known family history of morphea or other autoimmune disease. In April 2023, she experienced the onset of both swelling and a rash on her right lower leg after returning from Europe. A biopsy in September 2023 confirmed a diagnosis of morphea after initially being worked up for stasis dermatitis. At a visit in October 2023 with her initial dermatologist, the patient presented with numerous fibrotic plaques located on the upper extremities bilaterally, forehead, and trunk, with concomitant nail dystrophy on the right lower extremity. Her initial treatment regimen consisted of prednisone. While this temporarily halted the disease progression, it was ultimately discontinued due to the intolerance of side effects. Methotrexate was introduced thereafter, with a reported dosage of 15 mg per week. However, despite being on methotrexate for several months, she began to develop new plaques. The dose of methotrexate was later increased to 20 mg per week in March 2024, and no adverse effects from the medication were reported. However, due to the continued evolution of the plaques, she decided to discontinue methotrexate in April 2024.

Shortly thereafter, she moved to the South Texas region and sought to establish care with local rheumatology, wound care, and dermatologic specialists. Laboratory findings from an initial workup in June 2024 included both a positive anti-nuclear antibody (ANA) titer (1:1280) and positive rheumatoid factor (RF) (Table 1). 

**Table 1:** Laboratory findings for both ANA and RF ANA: Anti-nuclear antibody, RF: Rheumatoid factor

Laboratory studies	Result	Reference range
ANA screen immunofluorescence assay	Positive	Negative
ANA titer 1: Nuclear, nucleolar	1:1280	<1:40 (negative)
ANA titer 2: Nuclear, homogeneous	1:320	1:40-1:80 (low antibody level); >1:80 (elevated antibody level)
Rheumatoid factor	77 IU/mL	< 20 IU/mL

The patient established care at our UT Health Rio Grande Valley (UTRGV) dermatology specialty clinic (Edinburg, TX, USA) seeking further management options. During her first visit in June 2024, the patient presented with the chief complaint of both new and evolving skin plaques across her body, with progression of the ulcers located on her right lower leg. One plaque on her forehead resembled the ECDS subtype of linear morphea (Figures 1-6).

**Figure 1:**
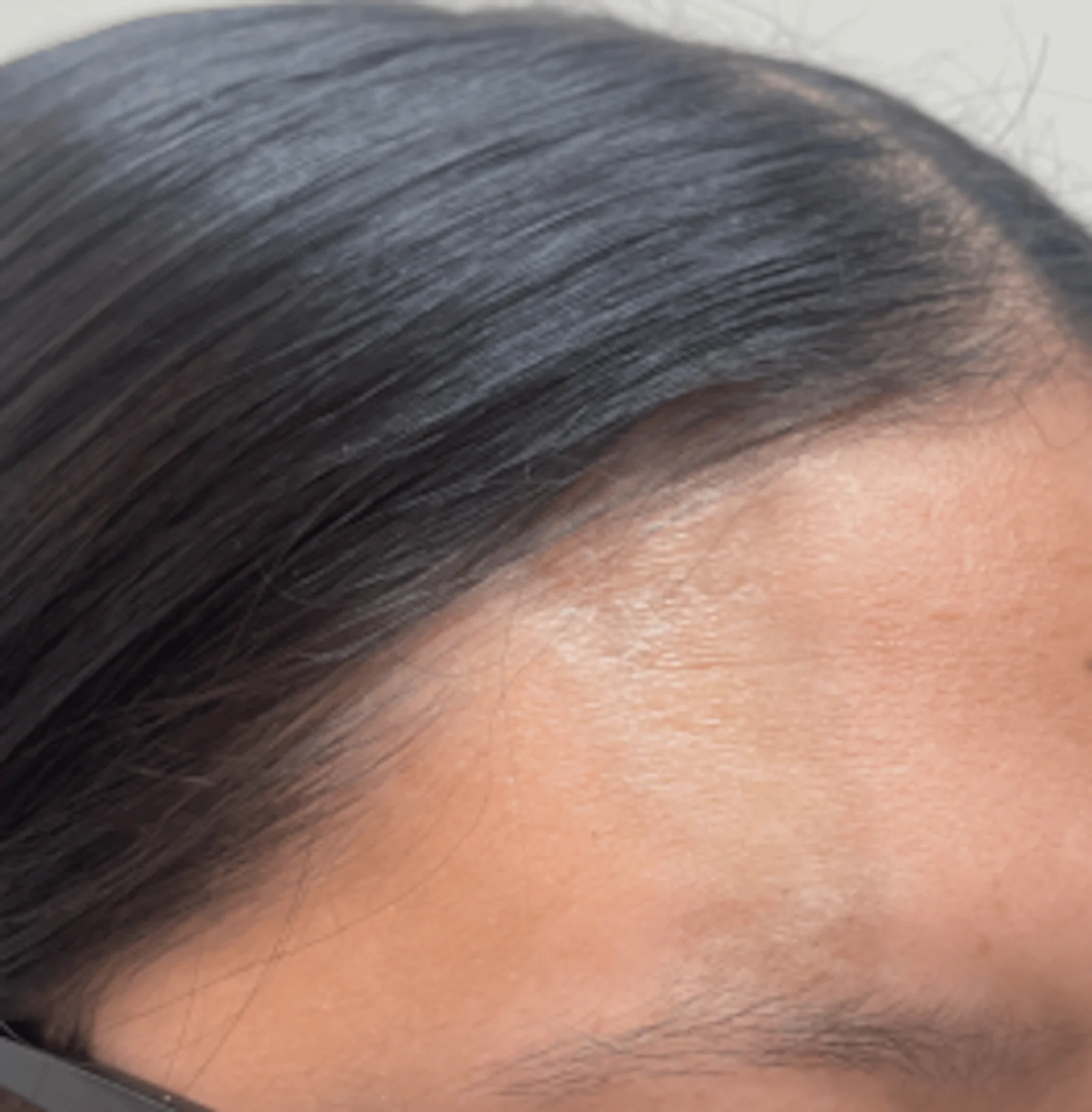
A linear, well-demarcated plaque on the right forehead extends towards the scalp The plaque is consistent with the ECDS subtype of linear morphea that may present as a linear induration on the forehead. ECDS: En coup de sabre

**Figure 2:**
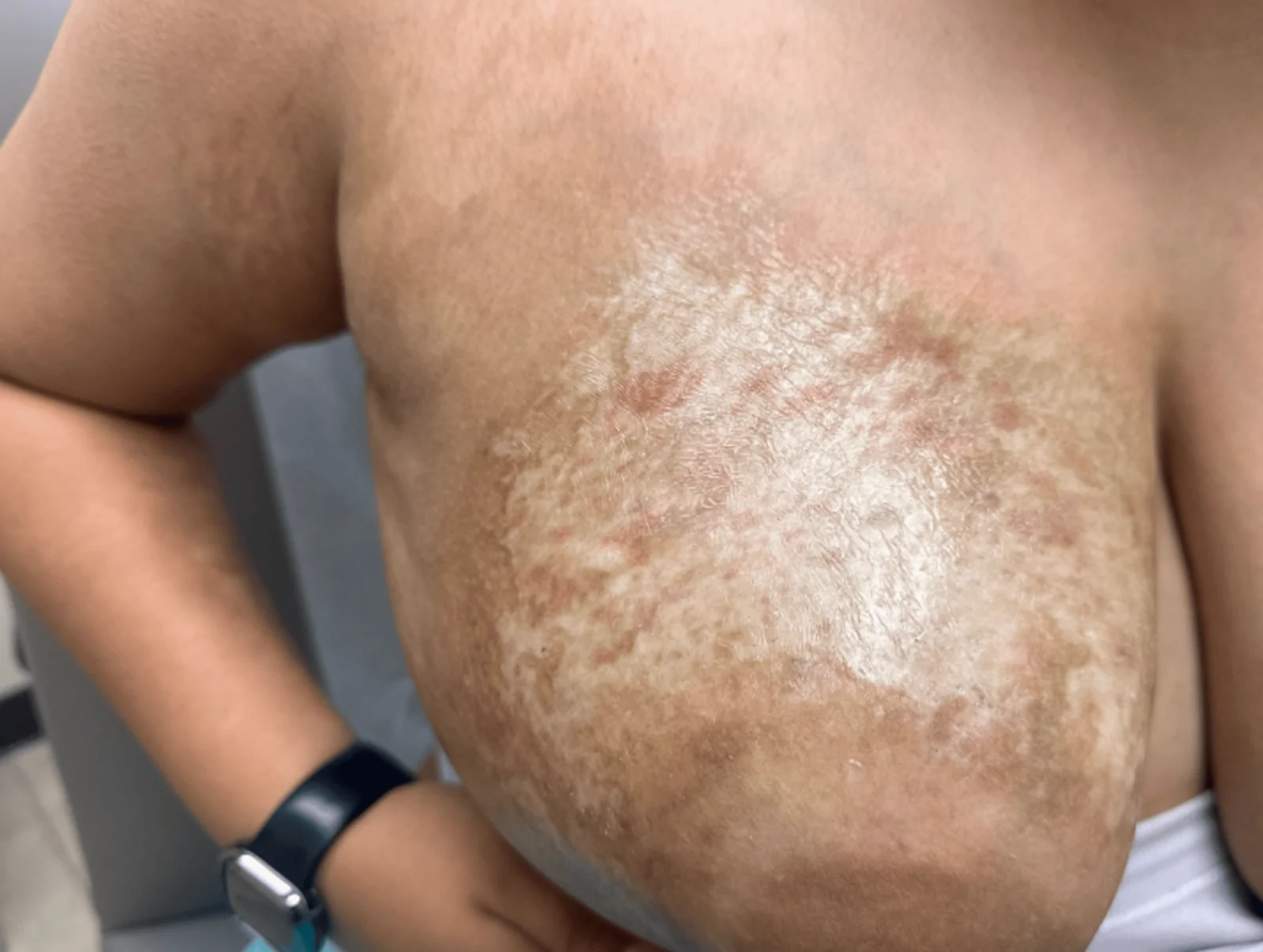
A sclerotic plaque with a rim of hyperpigmented patches on the right breast

**Figure 3:**
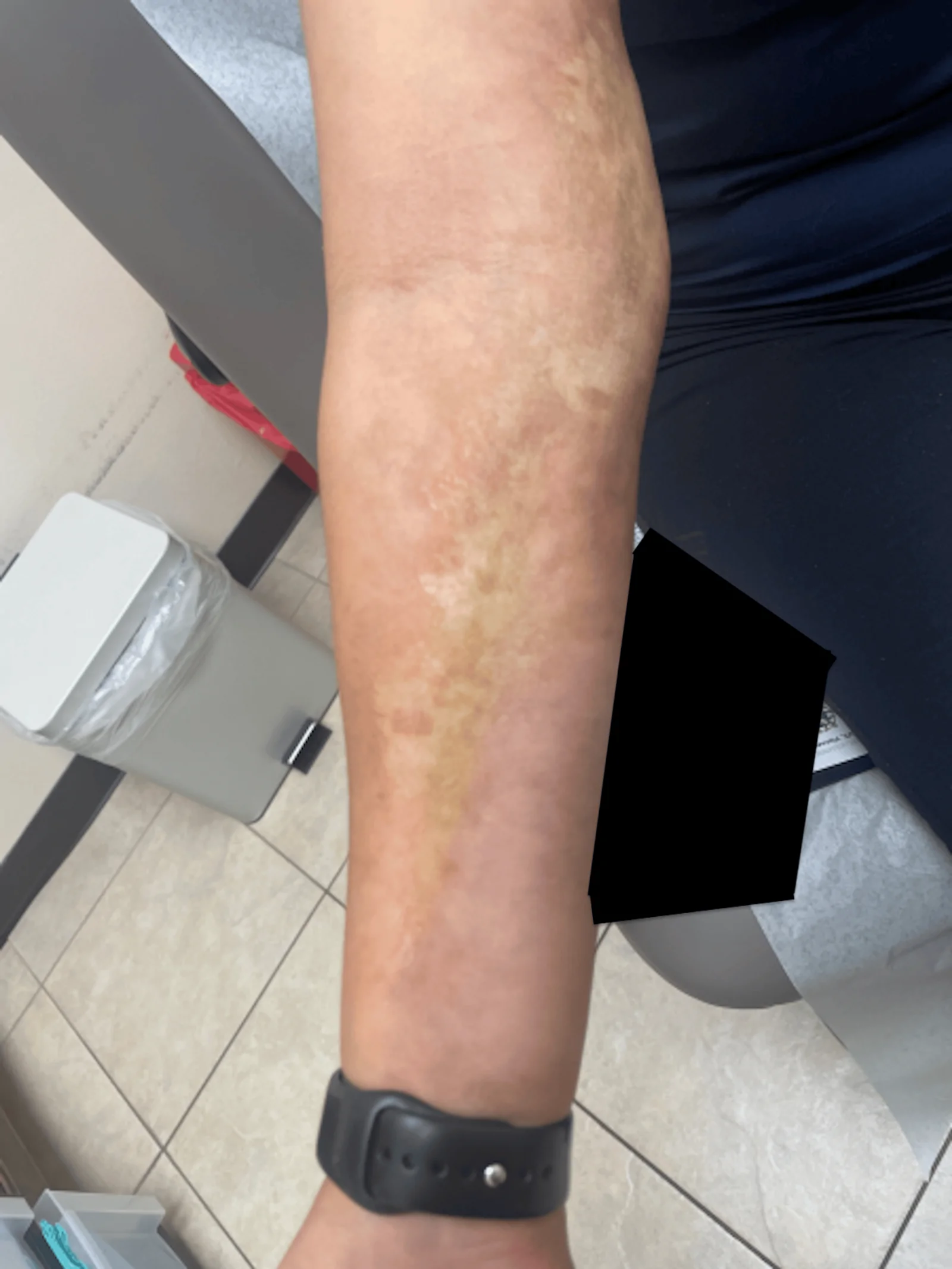
A sclerotic plaque with a rim of hyperpigmented patches on the right upper extremity

**Figure 4:**
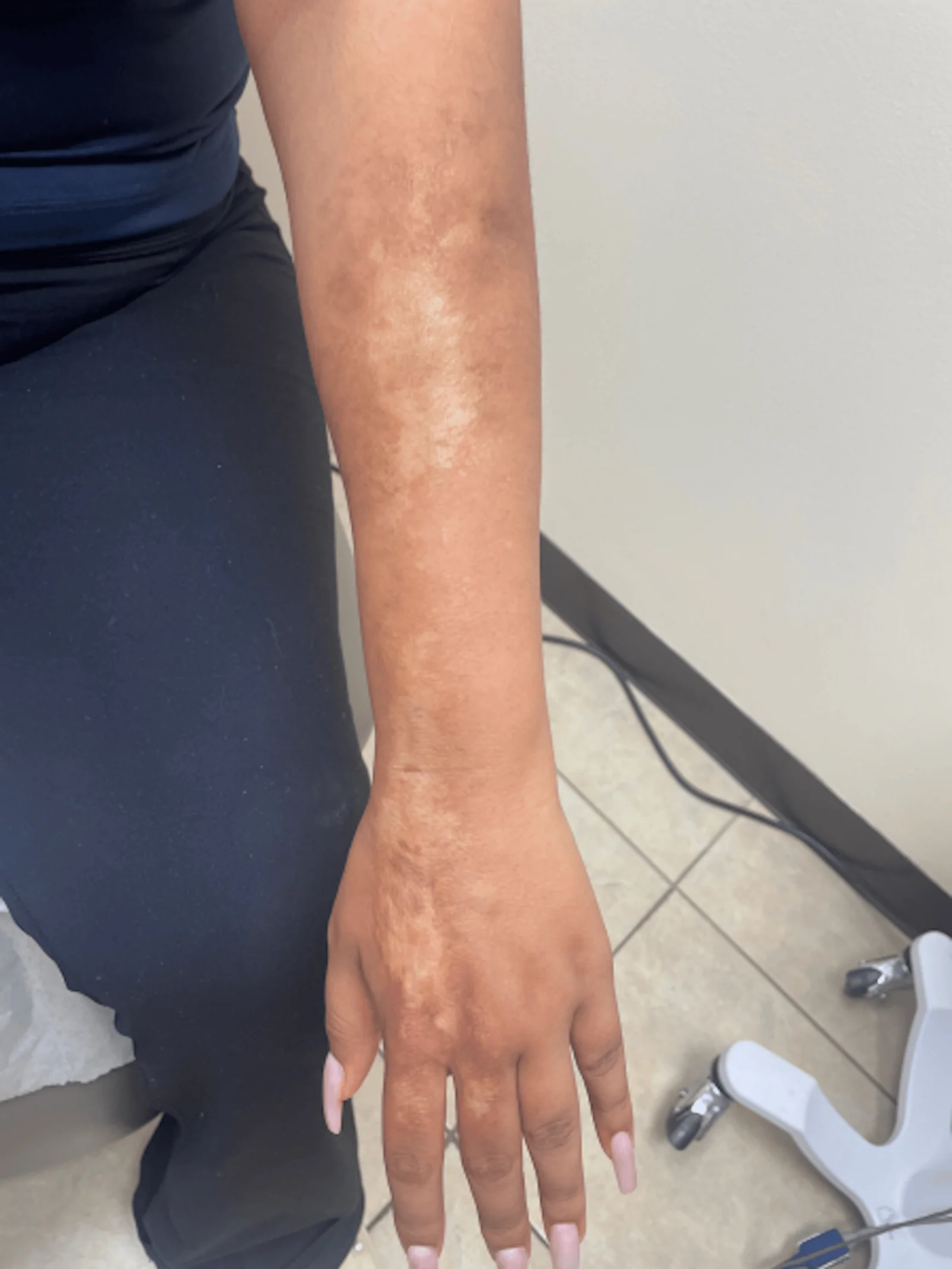
A sclerotic plaque with a rim of hyperpigmented patches on the left upper extremity

**Figure 5:**
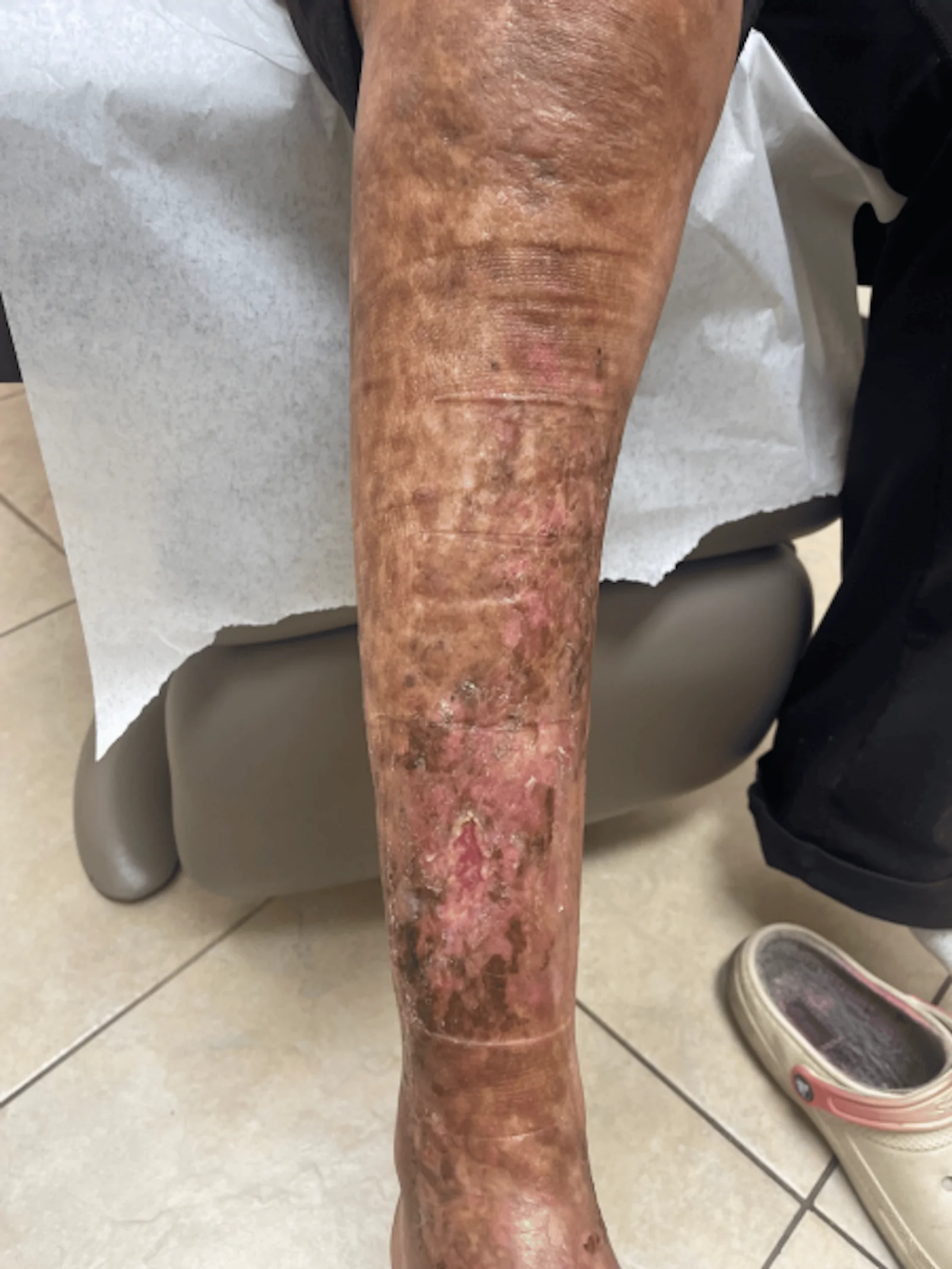
Numerous sclerotic plaques with a rim of hyperpigmented patches and ulcers observed on the right lower extremity

**Figure 6:**
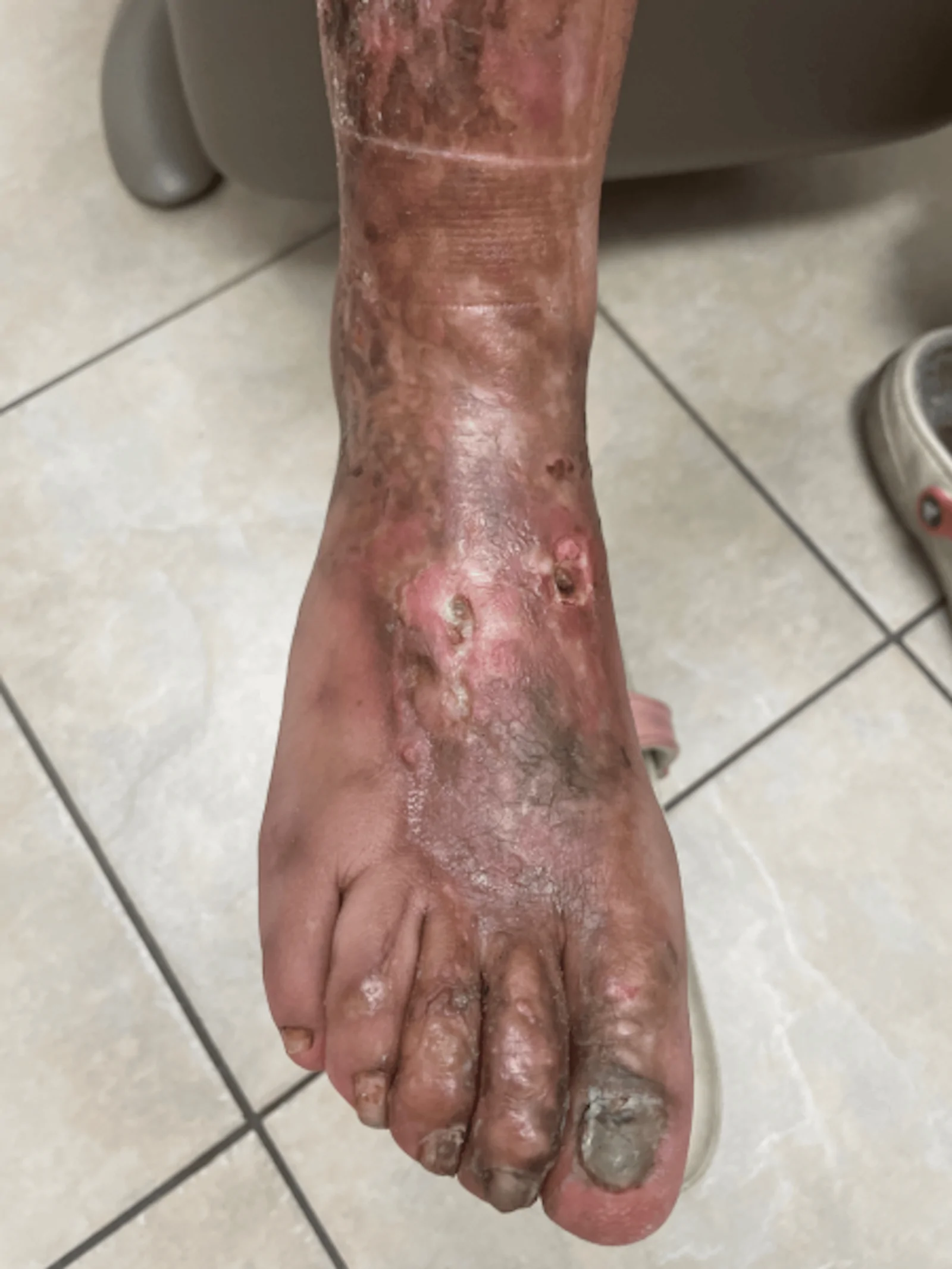
Numerous sclerotic plaques with a rim of hyperpigmented patches and ulcers observed on the right lower extremity

The patient expressed a strong preference to avoid systemic steroids due to prior side effect intolerance, such as weight gain. After further consideration, she was prescribed CellCept (mycophenolate mofetil), an immunosuppressant, 500 mg to take twice a day orally. Two topical medications were also prescribed for application on the right lower extremity: a clobetasol 0.05% and gentamicin 0.1% topical ointment. Finally, the importance of frequent follow-up with a wound care specialist for the lower extremity ulcers was emphasized during this visit.

The patient established care with a local rheumatologist in July 2024 to review her June laboratory studies. At this time, she had been tolerating the medication well but noted the onset of a new lesion located on her upper neck. Ultimately, recommendations were made to increase the CellCept dose to 1000 mg orally twice a day. Additional laboratory testing was obtained during this visit to ensure the patient qualified for the increased dosage (Table 2).

**Table 2:** Results from laboratory studies conducted during patients visit to the rheumatologist

Laboratory studies	Result	Reference range	Units
Albumin	3.7	3.5-5.0	g/dL
Alkaline phosphatase	86	40-150	U/L
Alanine transaminase (serum glutamic-pyruvic transaminase)	21	0-55	U/L
Aspartate aminotransferase (serum glutamic-oxaloacetic transaminase)	16	5-34	U/L
Basophile, alternate %	0.4	0.1-1.2	%
Basophile, alternate absolute (ABS)	0.02	0.01-0.08	10.e3/uL
Calcium	9.4	8.4-10.2	mg/dL
Chloride	108	98-107	mmol/L
Creatinine	0.72	0.57-1.11	mg/dL
Direct bilirubin	0.101	0-0.5	mg/dL
Eosinophil, alternate %	3.5	0.7-5.8	%
Glucose	94	70-99	mg/dL
Hematocrit	39.4	34.1-44.9	%
Platelets	256	182-369	10.e3/uL
Phosophate	3.9	2.3-4.7	mg/dL
Potassium	3.6	3.5-5.1	mmol/L
Protein, total	7.5	6.4-8.3	g/dL
Sodium	141	136-145	mmol/L
Total bilirubin	0.254	0.2-1.2	mg/dL
Urea nitrogen	12	7-18.7	mg/dL
WBC	4.55	3.98-10.4	10.e3/uL

At her first follow-up visit (August 2024) to the UTRGV dermatology specialty clinic, the patient reported the following: minimal progression of the plaques that were present at her visit in June, mild improvement of her lower extremity ulcer under the assistance of the wound care clinic, and the plan by her rheumatologist to increase the CellCept dose.

At her second follow-up visit (September 2024) at the UTRGV dermatology specialty clinic, the patient reported that her rheumatologist initiated a new medication: Plaquenil (hydroxychloroquine) 200 mg orally once a day. She endorsed tolerance to both the increased CellCept dose and Plaquenil concomitantly. Despite reporting mild improvement in the ulcers located on her right leg, she noted possible new plaque development on both the left lower extremity and under her chin. Likewise, she reported that her rheumatologist recently referred her to a morphea specialist in Dallas, Texas.

She was seen once more (third follow-up visit in November 2024) by the UTRGV dermatology specialty clinic. At this visit, the patient reported mild improvement in the sclerotic plaques and ulceration found on her right lower extremity (Figure 7). 

**Figure 7:**
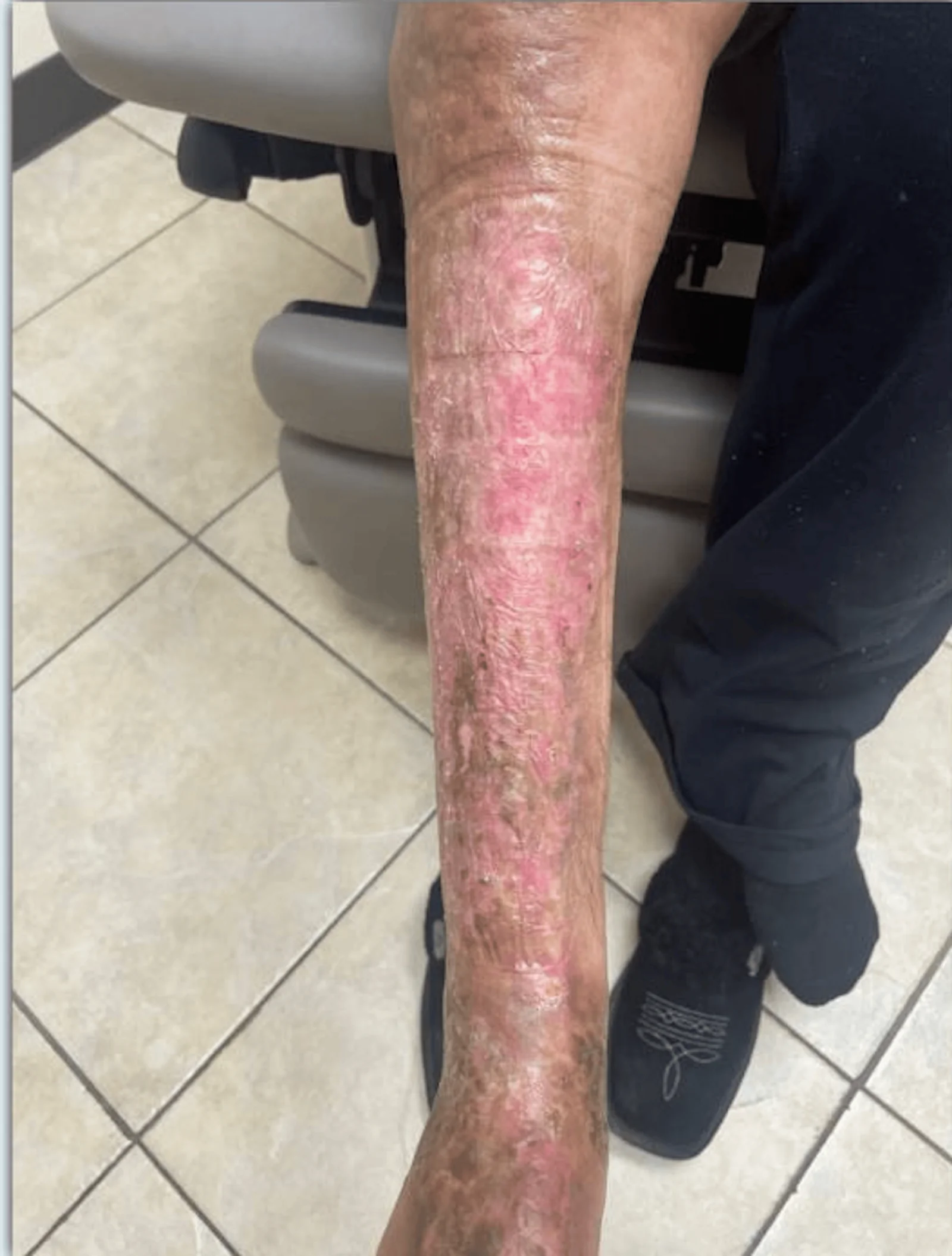
Gradual resolution of the sclerotic plaques and ulceration located on the right lower extremity

Following her referral to a morphea specialist, the patient established care with Dr. Heidi Jacobe at UT Southwestern Medical Center (Dallas, TX, USA) in January 2025. The patient was transitioned to a prednisone taper, restarted at 40 mg and tapered monthly to 20 mg, 10 mg, and 5 mg, in combination with methotrexate. This marked a new change to her medication regimen given continued disease activity despite CellCept and Plaquenil. At her fourth follow-up visit to our clinic (February 2025), her plaques were largely stable, the previously noted right lower extremity ulceration had resolved, and methotrexate was continued at 12.5 mg weekly. Two new musculoskeletal findings prompted an occupational therapy referral: early curvature of the right second toe with pain on ambulation and new finger stiffness.

The patient followed up with Dr. Jacobe in Dallas in March 2025. Given persistent concern for deeper tissue involvement, an MRI of the bilateral thighs was obtained; the MRI showed moderate subcutaneous atrophy, mild peri-fascial edema, and atrophy of the right gracilis muscle belly with enhancement at the myotendinous junction. These findings were consistent with disease activity extending into the fascia and muscle rather than the skin alone. While her cutaneous disease was overall less active than prior, her skin lesions spanned both a Blaschkoid distribution (consistent with linear morphea) and a more diffuse distribution. Due to this, the treating specialist favored an overall diagnosis of generalized morphea. Given inadequate response despite prior therapies, her regimen was transitioned to methotrexate as the primary agent, increased to 20 mg weekly, with a continued prednisone taper; she reported a 40-pound weight gain attributed to corticosteroid use. Unfortunately, the patient has been lost to follow-up since April 2025.

## Discussion

The cutaneous findings of morphea often present as erythematous, bruise-like lesions with sclerotic changes that extend to the epidermis alone or to the deeper dermal layers; deeper lesions are more likely to present as edematous, erythematous plaques with poorly defined borders [5]. The pathogenesis primarily involves profibrotic cytokines, such as IL-4 and TGF-β, which influence the activation of fibroblasts and excess collagen deposition [6].

Histologically, morphea is characterized by thickened dermal collagen that may subsequently evolve into a dense, compact region with loss of adnexal structures in established disease. Since it is difficult to distinguish morphea from systemic sclerosis (SSc) with routine histology, diagnosis remains primarily clinical [6]. Features favoring SSc include a mask-like facial appearance, Raynaud phenomenon, sclerodactyly, digital pitting or ulcers, and positive anti-centromere or anti-Scl-70 antibodies, along with internal organ involvement, none of which our patient demonstrated [6]. The patient's positive ANA and RF, while nonspecific, are a common finding in morphea and have been associated with a higher risk of deeper tissue involvement and even relapse [6].

Morphea has five distinct classifications: localized, generalized, linear, deep, and mixed [4]. Our patient met the criteria for a diagnosis of mixed morphea, as she presented with findings consistent with both the linear and generalized classifications. En coup de sabre, a subtype of linear morphea, requires the presence of a forehead lesion similar to the plaque found during our patient’s initial physical examination [4]. Likewise, the generalized classification may be diagnosed if there are four or more plaque-like lesions that measure up to three or more centimeters and appear on at least two or more anatomic locations [4]. Once again, our patient met these criteria based on the initial physical examination. In the literature, ECDS morphea and ECDS scleroderma are used interchangeably. Subtype classification can also evolve, as later observed in this patient by the treating morphea specialist, reinforcing that mixed morphea is best reassessed longitudinally. 

The medical implications for each morphea classification are variable. For example, mixed morphea is highly associated with a family history of autoimmune disease [4]. En coup de sabre is relatively more common in pediatric populations [7]. Some reports have suggested that pediatric cases of ECDS experience a higher rate of extracutaneous manifestations when compared to adult cases, though this could be attributed to insufficient adult data [7]. Concomitant neurological complications are common, with symptoms of seizure, stroke, and headaches having frequently been reported in patients with ECDS [7]. Thus, neuroimaging should be considered in ECDS patients to identify if any lesions are present within the central nervous system [8]. While our patient had not experienced any neurologic symptoms at the time of her visit to our clinic, neuroimaging was considered.

The first-line therapy for morphea may involve a combination of systemic corticosteroids and methotrexate [4,9]. In this case, prednisone provided early control but was discontinued due to side effects. Thereafter, methotrexate monotherapy was introduced but was not satisfactory. This prompted the switch to CellCept by the time the patient visited our clinic, as it is well tolerated in methotrexate-refractory cases [6]. Mycophenolate mofetil is considered a second-line treatment for both pediatric and adult morphea. It is typically reserved for cases that are either refractory or intolerant to methotrexate or severe relapsing disease, with retrospective cohorts supporting its efficacy and safety in this setting [6]. Plaquenil, an antimalarial that modulates immune activity to reduce inflammation without behaving as a direct immunosuppressant, was later added to our patient's regimen as a steroid-sparing adjunct consistent with the patient's preferences [10]. There are no current guidelines that specifically address adult morphea, so these choices are based on recommendations pertaining to pediatric localized scleroderma [9].

In morphea, cutaneous improvement does not rule out the potential for deeper disease activity: despite visible improvement of her leg ulceration, imaging obtained roughly a year into treatment revealed ongoing fascial and muscular involvement. This is consistent with reports that linear morphea over joints can cause contractures and limb-length or circumference discrepancies [6]. This case also highlights how adults must undergo ongoing screening for musculoskeletal complications, reflected here by her new toe and finger findings, and that refractory or deep disease benefits from sustained multidisciplinary coordination with dermatology, rheumatology, wound care, and occupational therapy rather than a single treatment trial.

## Conclusions

This case highlights the following: first, the variability in the clinical presentation of adult-onset morphea. Our patient presented with findings consistent with a mixed classification, which included both the generalized and ECDS variants. Second, a stepwise therapeutic approach towards a standard young adult patient by both dermatologic and rheumatology specialists. Finally, considerations for medication tolerance and outcomes. Due to our patient's concerns with both prednisone and methotrexate, she began CellCept therapy in June of 2024 with the UTRGV dermatology specialty clinic. Shortly thereafter, Plaquenil was added to her current regimen by a local rheumatologist. Fortunately, at her follow-up appointment with us in November 2024, she reported mild improvement of the ulcers located on her right leg. She subsequently established care with a morphea specialist in Dallas, Texas, where an MRI obtained in March 2025 revealed subcutaneous, fascial, and muscular involvement despite visible improvement of her cutaneous disease, prompting a transition to methotrexate as her primary systemic agent. Since March 2025, the patient has remained under multidisciplinary care with dermatology, rheumatology, wound care, and occupational therapy, with new musculoskeletal complications prompting additional intervention. This case reinforces the value of longitudinal care guiding long-term management of mixed and generalized morphea.
